# Clustering, hierarchical organization, and the topography of abstract and concrete nouns

**DOI:** 10.3389/fpsyg.2014.00360

**Published:** 2014-04-28

**Authors:** Joshua Troche, Sebastian Crutch, Jamie Reilly

**Affiliations:** ^1^Department of Speech, Language, and Hearing Sciences, University of FloridaGainesville, FL, USA; ^2^Department of Neurodegenerative Disease, Dementia Research Centre, Institute of Neurology, University College LondonLondon, UK; ^3^Eleanor Saffran Center for Cognitive Neuroscience, Temple UniversityPhiladelphia, PA, USA; ^4^Department of Communication Sciences and Disorders, Temple UniversityPhiladelphia, PA, USA

**Keywords:** semantic memory, concreteness, abstract concepts, embodied cognition, emotion, magnitude

## Abstract

The empirical study of language has historically relied heavily upon concrete word stimuli. By definition, concrete words evoke salient perceptual associations that fit well within feature-based, sensorimotor models of word meaning. In contrast, many theorists argue that abstract words are “disembodied” in that their meaning is mediated through language. We investigated word meaning as distributed in multidimensional space using hierarchical cluster analysis. Participants (*N* = 365) rated target words (*n* = 400 English nouns) across 12 cognitive dimensions (e.g., polarity, ease of teaching, emotional valence). Factor reduction revealed three latent factors, corresponding roughly to perceptual salience, affective association, and magnitude. We plotted the original 400 words for the three latent factors. Abstract and concrete words showed overlap in their topography but also differentiated themselves in semantic space. This topographic approach to word meaning offers a unique perspective to word concreteness.

## Introduction

A narrow empirical focus on concrete words yields an incomplete picture of the mental lexicon. Today, substantial gaps persist in our knowledge of the cognitive and neural underpinnings of abstract words (e.g., love, truth). Readers of English encounter abstract and concrete words with comparable frequency (Reilly, 2005; Reilly and Kean, 2007). Thus, it is difficult to justify sidestepping the abstract half of the lexicon that poses an empirical challenge.

Despite lopsided attention to concrete words, cognitive science has shown longstanding interest in abstract words (Locke, 1685). Empirical work in abstract-concrete word differences advanced rapidly in the late 1960s when psycholinguists defined *concreteness* and devised a means of measuring its strength. Concreteness, the extent to which a word can be perceived through the senses, is typically measured as a continuous, ratio level variable anchored by a zero point, with zero indicating no evoked perception (Paivio et al., 1968). Psycholinguists have compiled concreteness ratings for many thousands of words across numerous languages with the aim of elucidating the word concreteness effect, a term that reflects the collective advantage for concrete words in a variety of domains, including recall accuracy (Walker and Hulme, 1999), age of acquisition (Gilhooly and Logie, 1980), word list memory (Allen and Hulme, 2006), naming latency (Bleasdale, 1987), word recognition (Schwanenflugel et al., 1988), and dissociations in performance associated with neurological injury (Warrington, 1975, 1981; Breedin et al., 1994; Franklin et al., 1995; Bonner et al., 2009; Jefferies et al., 2009).

It has proven exceptionally difficult to develop a comprehensive theory accounting for the word concreteness effect (Connell and Lynott, 2012). Abstract and concrete words differ on a variety of non-semantic dimensions, including sound structure and morphological complexity (Reilly and Kean, 2007; Westbury and Moroschan, 2009; Reilly et al., 2012), polysemy and homonymy (Anderson and Nagy, 1991; Crutch and Jackson, 2011). Thus, when one observes a concreteness advantage in a particular task, it is not always clear where the locus of the effect lies (for an example see Kroll and Merves, 1986).

An intimate link between language and abstract word representation forms the backbone of today's dominant model of word concreteness. Paivio's (1991) Dual Coding Theory (DCT) offers a multiple semantics approach to word meaning based on the premise that verbal knowledge and visuoperceptual knowledge reflect two parallel but also highly interactive codes that support a word's meaning. Concrete words benefit from the support of both visual and verbal codes (i.e., they are dually coded), whereas abstract word meaning is mediated almost exclusively through a verbal code. DCT has proven its durability as a model that accounts for word concreteness effects in early childhood language learning and reading, as well as in neurological dissociations in adults (Franklin et al., 1994, 1995; Sadoski and Paivio, 2004; Sadoski, 2005).

Although DCT is compelling in scope, many psycholinguists now recognize the need for finer-grained specificity in delineating the topography of abstract and concrete words. Several approaches to concrete-abstract word representation have recently emerged to address this need. Gallese and Lakoff (2005) and Kousta et al. (2011) have proposed “embodied” approaches to abstract word representation that anchor abstract word meaning in somatic states such as emotion. These embodied approaches offer a radical departure from the dominant view that abstract words are mediated exclusively through symbolic, propositional knowledge. In one such approach, Kousta et al. (2011) argue that emotion is a powerful latent factor (with somatic and perceptual underpinnings) that underlies the meaning of abstract words (Andrews et al., 2009; Kousta et al., 2009, 2011; Newcombe et al., 2012). Kousta et al. further argued that many past studies of concreteness have confounded the constructs of imageability (i.e., the ability to evoke a mental image) and context availability and that when such confounding factors are tightly controlled, the concreteness advantage either disappears or modestly reverses such that abstract words show a processing advantage (but see Paivio, 2013).

Other theorists attribute abstract-concrete differences to the rapid access to contextual information for concrete words (i.e., context availability) (Schwanenflugel and Shoben, 1983), a greater number of semantic units to support concrete concepts (Plaut and Shallice, 1993) or greater number of semantic predicates for concrete items (Jones, 1985). An alternative formulation has suggested that abstract words have a relatively greater reliance upon associative information, whilst concrete words have a relatively greater reliance upon semantic similarity information (Crutch and Warrington, 2005). The predictions of this “different representational frameworks” hypothesis have been confirmed by a number of recent studies (Duñabeitia et al, 2009), with semantic similarity and association demonstrated to exert a graded effect across the concreteness spectrum (Crutch and Jackson, 2011).

Language researchers have long recognized the role of taxonomic hierarchies in concrete word representation (Rosch, 1973; Lakoff, 1990). For example, *dog* is a basic level concept that has both superordinate (e.g., *animal*) and subordinate distinctions (e.g., *collie*). Much of our knowledge of lexical category structure is derived from studies where participants generate lists of features (e.g., *dog* → *tail*) or associations (e.g., *dog* → *leash)* for concrete target words (Garrard et al., 2001, 2005; Cree and McRae, 2003; Rogers et al., 2004; Cree et al., 2006; Dilkina and Lambon Ralph, 2012). These feature listings yield distance metrics that speak to the family resemblance among concrete words. While these feature listing methods have some utility when applied to abstract words there are inherent weaknesses to this approach for abstract words. Abstract concepts, by their nature, lack the taxonomic hierarchical organization and unambiguous contextual properties imbued within concrete concepts and which make a feature listing method ideal (But see Barsalou and Wiemer-Hastings, 2005; Wiemer-Hastings and Xu, 2005 for examples of feature listing approaches for abstract concepts).

Recently a novel abstract concept feature (ACF) rating approach has been used in combination with multi-dimensional scaling techniques to examine distance metrics and cohesion among abstract words. This approach, developed by Crutch et al. (2012a,b), asks participants to rate the importance of particular types of information for the meaning of a concept. Crutch et al. originally performed this procedure on a corpus of 50 abstract words, spanning nine cognitive dimensions, including emotion, magnitude, and spatial relations. Unlike standard measures of word concreteness, this unique clustering solution revealed that concepts such as VAPOR and ILLUSION aggregate closely within semantic space. Standard semantic distance metrics gleaned through feature listing approaches or unidimensional ratings often fail to capture such similarities.

Here we performed the ACF in order to determine the clustering attributes of larger corpus of concrete and abstract concepts within a higher dimensional space than was originally employed by Crutch et al. (2012a,b). We measured each word's salience on 12 unique dimensions, including: Sensation, Action; Thought; Emotion; Social Interaction; Time; Space; Quantity; Polarity; Morality; Ease of Modifying; and Ease of Teaching.

Sensorimotor information has long been known to play an important role in the representation of concrete concepts, and a growing body of research has made the argument for the role of affective association in the representation of abstract concepts (Andrews et al., 2009; Kousta et al., 2009, 2011). We included metrics for *Sensation, Action, Emotion*, and *Polarity* based on the dominance of these variables in previous work. We also included a more nuanced set of dimensions linked to *Social Interaction* and *Thought. Our rationale for the inclusion of these* dimensions stems from the work of Borghi et al. (2011) and Barsalou (1999), who argue for the contributions of social interaction and introspection on abstract word acquisition and representation. We assessed the salience of *Time* in abstract and concrete word meaning due to its role in the temporal unfolding of event structure (Allman and Meck, 2012). We assessed the salience of Spatial information due to its roles both in the organization of geographical concepts, as well as more oblique contributions to metaphor (Zwaan and Yaxley, 2003; Lakoff and Johnson, 2008) We assessed *Quantity* with the aim of tapping the division between numerical and non-numerical semantics (e.g., mass-count distinctions) (Gathercole, 1985). The *Morality* dimension characterizes the social mores that govern behavior which have been hypothesized to reflect a cognitive emotional association complex which can be represented across the prefrontal cortex and limbic system (Moll et al., 2005). *Ease of teaching* reflects variety in both age of acquisition and learning style (e.g., experiential observation vs. explicit verbal instruction) that mark abstract and concrete words (Coltheart et al., 1988; Strain et al., 2002; Reilly et al., 2007). *Ease of Modifying* provides an index of the contextual availability of a word in terms of adjectival description (Schwanenflugel and Shoben, 1983; Schwanenflugel et al., 1992). It should be noted that this is not an exhaustive list of dimensions and that the inclusion of certain dimensions is more empirically/theoretically justified than others. It should also be noted that we were constrained by selecting dimensions that could be easily distinguished and comprehended by the lay participant.

### Hypotheses, aims, and significance

The DCT is premised upon the interaction of two parallel semantic memory systems, one dedicated to sensory imagery and the other dedicated to language. We hypothesize that word concreteness might ultimately be better contextualized within one semantic system. One might specify such a system in terms of a high dimensional space where word meanings cluster along axes representing key cognitive dimensions (e.g., emotional salience, sensory salience). We hypothesize that this unitary space comprises a topography wherein the meanings of words (both concrete and abstract) are distributed. Here, we investigated the clustering behaviors of a relatively large (*N* = 400) set of abstract and concrete nouns within a semantic space bounded 12 dimensions, including: Sensation; Action; Thought; Emotional Valence; Social Interaction; Time; Space; Quantity; Polarity; Morality; Ease of Modifying; and Ease of Teaching.

We hypothesize that this topographic approach would produce regions of overlap, as well as distinct clusters corresponding to “concreteness” (e.g., abstract words cluster at the high end of emotional valence). Importantly, the presence of a unitary, multi-dimensional space would obviate the need for an artificial dichotomy such as concreteness by treating this and other psycholinguistic variables as continuous.

## Methods

### Overview

We isolated a set of abstract (*N* = 200) and concrete (*N* = 200) English nouns and obtained Likert-scale ratings for each word on 12 variables (dimensions). We then employed factor reduction and hierarchical cluster analysis to model the topography of how these words scaled.

### Participants

Participants included native English speakers recruited through the online crowd-sourcing program, Mechanical Turk. Following trimming procedures aimed at eliminating spurious participants, we isolated a sample (*N* = 365) with an age ranging from 17 to 83 years, (mean = 40.7). Education ranged from 9 to 20 years (mean = 15.4). Sex distribution was 68.2% female.

### Materials and procedure

Stimuli included English nouns (*N* = 400) from the MRC Psycholinguistic Database (Coltheart, 1981). Stimuli were pure nouns in that we ensured they had no alternate grammatical class (e.g., *desk* but not *phone*). Target words were either abstract or concrete based on rated concreteness. The MRC database concreteness values reflect a 100–700 scale. In our sample, concrete words had an average concreteness rating of 589 (*SD* = 46.9), whereas abstract words had a rated average of 304 (*SD* = 47.1). There was no overlap in the distributions of abstract and concrete words, and their means were distant (*Z*_difference_ = 2.38). The list of dimensions chosen for the analysis was not an exhaustive set of dimensions. In order to provide proof of concept that this clustering procedure could prove successful, we sampled words from the tails of the concreteness spectrum (high/low).

### Scale development and implementation

Participants rated each of the target words on the following 12 dimensions using a 7-point Likert Scale: 1. Sensation; 2. Action; 3. Thought; 4. Emotional Valence; 5. Social Interaction; 6. Time; 7. Space; 8. Quantity; 9. Polarity; 10. Morality; 11. Ease of Modifying; 12. Ease of Teaching. Table 1 reflects the wording given to participants.

**Table 1:** **Parameter description**.

**Parameter**	**Definition**
Polarity	I relate this word to positive or negative feelings in myself
Sensation	I relate this word to physical feelings like vision, hearing, smelling, etc
Action	I relate this word to actions, doing, performing, and influencing
Thought	I relate this word to mental activity, ideas, opinions, and judgments
Emotion	I relate this word with human emotion
Social interaction	I relate this word with relationships between people
Time	I relate this word with time, order, or duration
Space	I relate this word to position, place, or direction
Quantity	I relate this word to size, amount, or scope
Morality	I relate this word to morality, rules, or anything that governs my behavior
Ease of modifying	I can easily choose an adjective for this word (the ugly truth, whole truth, etc.)
Ease of teaching	This word could be easily taught to a person who does not speak English

Each stimulus appeared in randomized order within the context of separate surveys dedicated to each cognitive dimension. Participants were instructed to use the entire scale and to work quickly but carefully.

### Data collection

Participants completed ratings via Amazon Mechanical Turk, an online pool of workers from around the globe who perform virtual tasks (Buhrmester et al., 2011). Participants logged into Mechanical Turk, electronically consented, and then completed up to 12 individual surveys, one for each dimension.

### Data analyses

We excluded participant data that corresponded to any of the following conditions: (1) Taking less than 10 min to complete the survey (less than 1.5 s per response), (2) Using less than half of the seven point scale (i.e., 3 numbers or less) which was considered not following our directions of using the entire scale, or (3) The presence of runs of more than 20 identical consecutive responses (2.5 *SD* away from the average run mean; *M* = 3.2, *SD* = 6.8). We then performed intraclass correlational analyses in order to measure inter-rater reliability. We also ran correlation analyses between individual item standard deviations and concreteness in order to determine if concreteness led to greater variability in the rating of items.

We first pursued exploratory factor analysis with the goal of reducing the dimensionality and redundancy of the original set of 12 variables. We converted the original ratings into a series of factor scores using the Anderson-Rubin method (Anderson and Rubin, 1956). The factor analyses yielded three latent factors that subsequently define a three-dimensional space upon which distance metrics between any two words can be derived. We report the Euclidean squared coefficient as a metric of semantic distance (Danielsson, 1980).

Using the reduced dataset, we then conducted a hierarchical agglomerative cluster analysis using Ward's method (1963). This procedure iteratively clusters observations into groups in a bottom-up manner until only one large cluster remains. We determined the optimal clustering solution by comparing clusters from the hierarchical cluster analysis with clusters created by a partitional k-means iterative analysis using Cohen's Kappa (Aldenderfer and Blashfield, 1984). The cluster analysis allowed us to create an empirical metric of how items grouped in the semantic. In other words this allowed us to determine how items grouped on a smaller dimensions as compared to macro dimensions (i.e., Abstract-Concrete).

## Results

### Data trimming

The first author and a blinded rater showed 99.3% inter-rater agreement on surveys to be excluded (see method for criteria). Of the original 545 surveys, 180 (33%) were eliminated, leaving 365 surveys for final analysis (See Supplementary Material for how many responses were removed per condition). Removal was comparable across all surveys. The intraclass correlation coefficient (ICC) was found to high throughout all 12 surveys with the lowest ICC being 0.991 (see Table 2). Table 3 displays the correlations between item standard deviations and concreteness for each survey dimension. Two of the dimensions showed greater variability for more concrete concepts, three showed no variability differences and seven showed greater variability for more abstract items.

**Table 2:** **Inter-rater reliability**.

**Parameter**	**ICC**
Space	0.996
Morality	0.995
Quantity	0.992
Social interaction	0.995
Ease of teaching	0.994
Sensation	0.996
Time	0.944
Action	0.991
Ease of modifying	0.996
Thought	0.993
Emotion	0.997
Polarity	0.996

**Table 3:** **Correlation of concreteness and dimension *SD***.

**Dimension**	**Correlation**
Action	0.09
Ease of modifying	−0.55^*^
Ease of teaching	−0.31^*^
Emotion	−0.57^*^
Morality	−0.36^*^
Polarity	−0.61^*^
Quantity	0.08
Sensation	0.06
Social interaction	−0.43^*^
Space	−0.24^*^
Thought	0.36^*^
Time	0.23^*^

* *α < 0.01*.

### Individual ratings emotion

**Figure 1:**
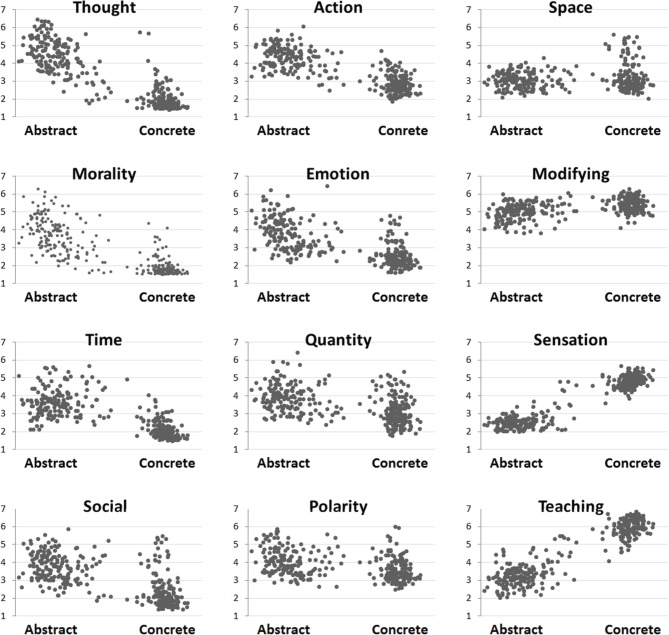
**Scatterplots of mean Likert scale ratings (1–7; y-axis) for each of the 12 rating dimensions for words from across the concreteness spectrum (x-axis)**.

Figure 1 reflects scatterplots of ratings for each of the 12 original dimensions plotted against the a priori concreteness values for each target word. All of the bivariate correlations were significant (α ≤ 0.01).

### Exploratory factor analysis

We extracted three latent factors (model fit, *R*^2^ = 0.81) from the original set of 12 dimensions (see Table 1). The reduced set of factors and the constituent variables they subsume were as follows: (1) Emotion, Polarity, Social, Morality, Action, Thought; (2) Ease of Teaching, Sensation, Ease of Modifying, Time; (3) Space, Quantity (see Table 4). In terms of nomenclature, we will refer to these latent constructs hereafter as: (1) Affective Association/Social Cognition; (2) Perceptual Salience; and (3) Magnitude.

**Table 4:** **Factor analysis/component matrix for dimensions**.

		**Component**	
**Predictor**	**Factor 1**	**Factor 2**	**Factor 3**
Emotion	0.905	0.229	−0.027
Polarity	0.880	−0.115	0.235
Social	0.855	0.280	0.090
Morality	0.794	0.479	0.057
Action	0.722	0.517	0.169
Thought	0.719	0.594	0.094
Ease of teaching	−0.376	−0.880	−0.040
Sensation	−0.447	−0.846	−0.026
Ease of modifying	0.104	−0.736	0.310
Time	0.350	0.685	0.319
Space	−0.006	−0.208	0.846
Quantity	0.273	0.412	0.691

*The above component matrix was derived using SPSS-18′s factor analysis algorithm employing a Varimax rotation with Kaiser normalization. The rotation converged after five iterations*.

**Figure 2:**
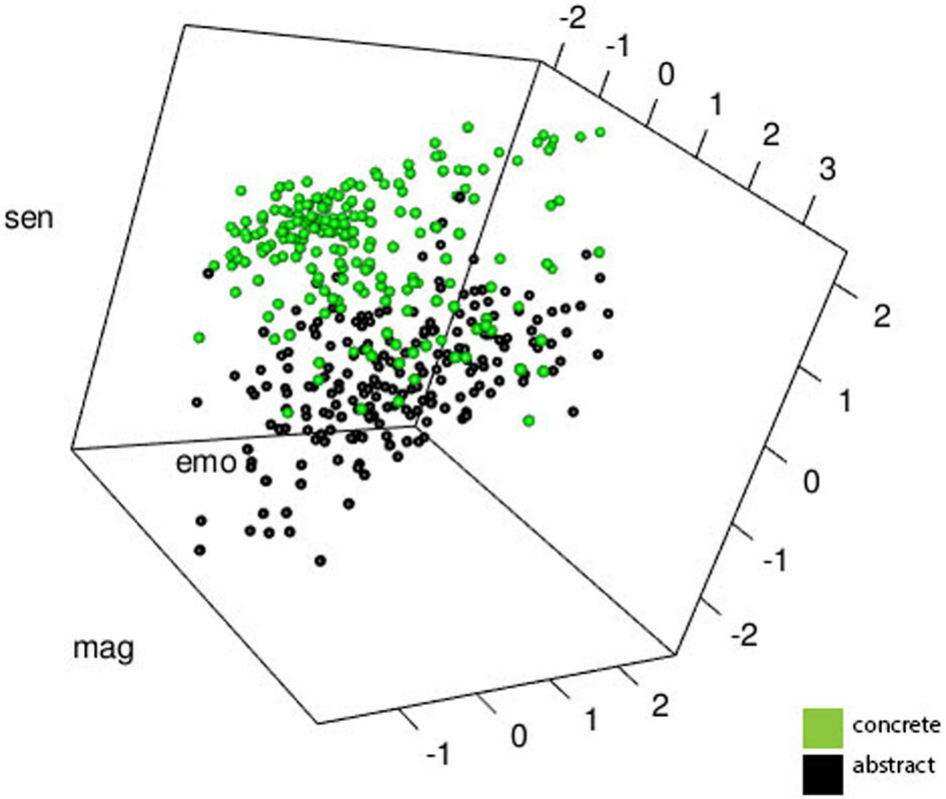
**Three Dimensional Scatterplot Representing Abstract and Concrete Word Meaning**. This view represents rotation about the axes/planes defined by the factors: Sens, sensation; Mag, magnitude; and Emo, emotion.

Table 5 represents relations between the three factors with other salient psycholinguistic variables (e.g., word frequency, age of acquisition). Figure 2 displays the spread between concrete and abstract words within the 3-dimensional space defined by the three factors.

**Table 5:** **Psycholinguistic and factor score correlation matrix**.

	**Imag**	**AOA**	**Frqy**	**CNC**	**Fam**	**Emo**	**Cnc/Tch**	**Mag**
Imag	1							
AOA	−0.86^*^	1						
Frqy	0.22^*^	−0.44^*^	1					
CNC	0.94^*^	−0.85^*^	0.21^*^	1				
Fam	0.29^*^	−0.56^*^	0.44^*^	0.27^*^	1			
Emo	−0.36^*^	0.23^*^	0.26^*^	−0.56^*^	0.16^*^	1		
Cnc/Tch	0.73^*^	−0.84^*^	0.40^*^	0.78^*^	0.51^*^	0	1	
Mag	−0.01	−0.03	0.09	−0.06	0.24^*^	0	0	1

Imag, Imageability; AOA, Age of Acquisition; Frqy, Frequency; CNC, concreteness; Fam, Familiarity; Emo, Emotion/Social Cognition; Cnc/Tch, Concreteness/Ease of Teaching; Mag, Magnitude;

* *p < 0.01*.

### Hierarchical cluster analysis

**Figure 3:**
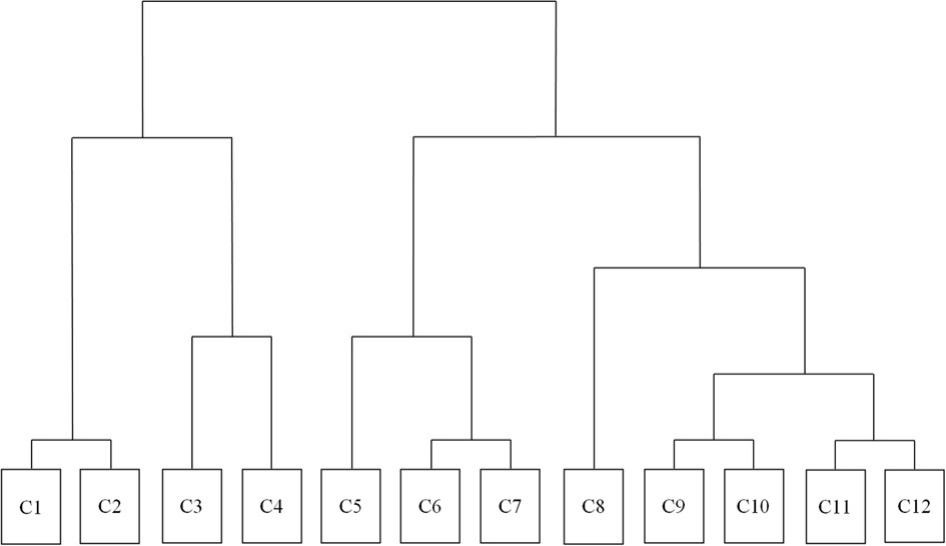
**Dendrogram of hierarchical cluster analysis**. Each cluster has been given a cluster number (e.g., C1,C2). The words inside each cluster can be found in Supplementary Material.

A 12-cluster solution yielded an optimal model (Cohen's Kappa = 0.87). Figure 3 reflects a dendrogram corresponding to this optimal clustering solution. Table 6 reflects quantitative aspects of each cluster in terms of psycholinguistic attributes (e.g., lexical frequency).

**Table 6:** **Psycholinguistic properties of clusters**.

	**C1**	**C2**	**C3**	**C4**	**C5**	**C6**	**C7**	**C8**	**C9**	**C10**	**C11**	**C12**
Imag	595.54	618.53	587.05	608.22	340.81	240.38	289.52	563.09	370.13	383.73	362.67	309.70
AOA	280.07	249.86	311.50	270.00	500.53	617.00	495.15	221.00	512.50	463.38	442.67	478.00
Frqy	14.05	53.98	38.85	59.71	6.14	1.48	9.37	223.05	7.40	23.50	45.46	22.97
CNC	598.67	605.60	585.65	588.21	326.73	285.67	301.10	574.48	272.25	303.00	312.53	310.64
Fam	519.33	564.40	534.70	557.06	485.19	424.43	505.00	584.95	465.63	529.27	557.59	528.48
Emo	−0.86	−0.15	−0.88	−0.65	−0.80	−0.34	0.33	1.23	1.34	1.67	1.62	0.60
Per	0.54	1.25	0.68	0.80	−1.59	−1.59	−0.95	1.69	−0.45	−0.02	0.05	−0.67
Mag	−0.85	−0.55	2.55	0.60	0.54	−1.48	−0.45	−0.30	−1.60	−0.17	0.99	0.65

*Imag, Imageability; AOA, Age of Acquisition; Frqy, Frequency; CNC, concreteness; Fam, Familiarity; Emo, Emotion/Social Cognition; Per, Perceptual Salience; Mag, Magnitude*.

The dendrogram shows that most concrete words are contained in the first four clusters (C1–C4), whereas abstract words are mostly found in latter clusters (C5–C12). Focusing on the clusters of abstract words, it is apparent that the level of affective association increases from left to right on the dendrogram. Cluster 8 is also of interest as it is a cluster of concrete words (e.g., chocolate, father) that are high in affective salience and nestled within many other abstract words describing social cognition.

## Discussion

Using hierarchical cluster analyses, we explored the topography of abstract and concrete nouns (*N* = 400). We first defined a multi-dimensional semantic space that was composed of 12 individual predictors, each with precedence as a moderator of concreteness effects. Participants subsequently rated the original set of abstract and concrete nouns on all of the individual dimensions. We then used factor analysis to examine whether the original multi-dimensional semantic space could be reduced. This approach yielded three latent constructs, corresponding roughly to affective association/social cognition, perceptual salience, and magnitude. We then calculated distance metrics for the abstract and concrete words within the semantic space defined by this reduced set of predictors. Abstract and concrete words have both unique and common regions of overlap within semantic space. Moreover, factors such as affective association/social cognition and magnitude appear to play significant roles in delineating this space.

There are two primary ways of visualizing these data. The first is at the level of the individual predictors, and the second is through a clustering analysis that considers the predictors together.

### Individual predictors

Figure 1 highlights the variability and weighting across the 12 unique dimensions in isolation prior to factor reduction. The bivariate correlations between concreteness and each predictor vary from strongly positive (e.g., *r* = 0.94 for *sensation*) to strongly negative (e.g., *r* = −0.87 for *thought*). In addition, several predictors (e.g., *r* = 0.10 for *space*) had relatively flat slopes, indicating that these variables only weakly discriminated concrete from abstract words in isolation. With respect to concreteness, we observed the strongest positive bivariate correlations with sensation (*r* = 0.94) and ease-of-teaching (*r* = 0.92). Sensation, analogous to imageability, is a construct intimately related to concreteness (*R*^2^ = 0.88) but one that captures a wider range of somatosensory states. Ease-of-teaching has a close parallel to ease of learning. A vast body of literature investigating age-of-acquisition has shown that the earliest acquired words tend be concrete (e.g., ball, mama). One common developmental explanation is that the salience of a concrete word's referent facilitates a fast and durable mapping (Gilhooly and Logie, 1980; Bloom, 1998). Abstract words, in contrast, have no physical referent and must therefore be learned through alternate means, often through nuanced experiences with concrete objects and emotions. For example, one must first learn “sad” before acquiring a more abstract state such as “melancholy.”

In addition to strong positive relationships with concreteness, we also observed several robust negative correlations, including *thought* (*r* = −0.87) and *morality* (*r* = −0.81). Participants rated *thought* according to the salience of ideas, opinions, judgments, and mental operations. Many words that are considered classically abstract are often defined as “the feeling of X.” Thus, the strong negative correlation between concreteness and *thought* reflects a logical property of abstract words (i.e., they tend to often denote unobservable mental states). *Morality* is similar to *thought* in that this construct often denotes phenomena that are not directly observable but instead reflect complex social mores that govern and denote behavior (e.g., truth, honesty).

### Multidimensional solution

The strength of this approach lies not within individual predictors but in a solution that considers all such variables simultaneously. This multi-dimensional solution yielded a dynamic structure whereby abstract and concrete words can be differentiated. We view two properties of the observed topography as particularly salient: (A) Abstract and concrete words have unique topographies within a multi-dimensional space defined by affective association/social cognition, magnitude, and perceptual salience; (B) The topography of abstract and concrete words also overlap within this space. For example, *father* and *love* load high on emotion and ultimately cluster together despite the fact that *father* is classically considered concrete and “love” as abstract. It should be noted that this clustering emerges despite all words being rate independently (i.e., there were no ratings of the direct association between any pairs of concepts).

#### The topographies of abstract and concrete words are unique

While affective association/social cognition and concreteness/perceptual salience have been regularly indicated as dimensions that underlie the representations of concrete and abstract concepts, the role of magnitude is less clear.

The factor analysis identified a latent variable reflecting a combination of space and quantity. We interpreted this amalgamation as corresponding roughly to the construct of magnitude. Magnitude in this context reflects both the scalar features of concrete words (e.g., how large?, how hot?, how loud?) but also gradations of many abstract emotions (e.g., irritated < angry < infuriated). Walsh (2003) has argued that such a magnitude system detects and appreciate such gradations. Neurological damage to regions of the parietal lobes (e.g., cortical basal degeneration) results in deficits for estimating and appreciating many magnitude distinctions, including time, physical size, and affect (i.e., emotional blunting; Gibb et al., 1989; Crutch et al., 2012a,b).

Magnitude is a construct that has previously received attention in the psycholinguistic literature, particularly with respect to spatial metaphor comprehension (Lakoff, 1990, 2012; Barsalou and Wiemer-Hastings, 2005; Jefferies et al., 2009; Connell and Lynott, 2012). During semantic relatedness tasks (e.g., match two related pictures from a field of three), both healthy adults and patients with neurological disorders (e.g., stroke aphasia) tend to take longer to match items that are more geographically distant (e.g., London:New York vs. London:Manchester; Crutch and Warrington, 2003), or items that appear in reverse-iconic order (e.g., basement:attic vs. attic:basement; Zwaan and Yaxley, 2003). Similar findings have been reported for the directionality and congruency of spatial metaphors with respect to one's own body (Zwaan and Taylor, 2006). Thus, our scaling results confirm a place of prominence and a dimension of discrimination for magnitude and related variables (e.g., polarity, valence) in supporting the meanings of both abstract and concrete words.

#### The topographies of abstract and concrete words also overlap

The scatterplot in Figure 2 demonstrates several regions of significant overlap in the topographies of abstract and concrete words. The area of highest overlap was apparent for words at the high end of the affective association/social cognition dimension. Concrete words that loaded high on the affective association/social cognition factor (e.g., father, chocolate) were closer via distance metrics in semantic space to abstract words (e.g., love, justice) than they were to other concrete or abstract words lacking an affective association/social cognition component (e.g., aspect, paradigm, fisherman, and banana). This underscores the importance of emotional valence in word meaning. Altarriba et al. (1999) have argued that emotional valence can be viewed as orthogonal to concreteness and should accordingly be viewed as an independent dimension of word meaning (i.e., there are abstract, concrete, and emotion words). More recently Kousta et al. (2011) have argued for an embodied theory with emotional information being the main contributor to the representation of abstract concepts (Etkin et al., 2006; Vigliocco et al., 2013).

The overlap of our topographies in areas of high affective association/social cognition suggest that while abstract concepts likely rely more on affective association/social cognition for their representation, concrete concepts can also be greatly influenced by affective association/social cognition. There is also the indication that high affective association/social cognition can lead to abstract concepts becoming more tangible, that is, more concrete, as indicated by the positive association between affective association/social cognition and imageability. This overlap may lead to a strengthening of the networks for these concepts leading to collective processing advantages that Kousta et al. (2009) found for words high in affective association. It should be noted that these areas of overlap are even more surprising as we only chose concepts that were at the extreme ends of the concreteness spectrum.

The ACF approach allowed us to create a single multidimensional semantic space. This approach obviates the need for multiple semantic systems (e.g., language for abstract words, percepts for concrete words). By treating this topography as a continuous space, word meaning can be distributed in a flexible way that is untethered to any particular artificial dichotomy (e.g., abstract-concrete, imageable-non-imageable; for another unitary semantics account see Vigliocco et al., 2004). In this approach words were rated individually, therefore words collocated in this semantic space represent similar underlying properties and not merely linguistic properties. It should be noted that early work on dimensionality in semantics by Osgood et al. Osgood et al. (1954) also found three dimensions that held importance in the evaluation of concepts: evaluation, potency, and activity. This work, however, has mostly focused on determining the connotation of a concept, object, or event.

It still remains an open question, however, whether this semantic space is neurologically real or just a product of our data. We attempted to test this question through the use of a behavioral task with a patient with aphasia (Crutch et al., 2013). The patient, a 65 years old male, had a history of global aphasia which resolved into a mixed non-fluent aphasia. This patient, SKO, displayed deficits in verbal comprehension and phonological-orthographic transcoding. The patient was given a spoken word to written word matching paradigm. This consisted of SKO being shown two words and then being asked to point to the word just spoken by the examiner. The pairs of words were varied by distance. Some of the words were close in distance in the semantic space created in the current study while others were far. As we had predicted, pairs of words closer in semantic distance lead to greater interference than those further. We also determined that ACF ratings were better at predicting deficits than another common and well researched method of determining the strength of word association, latent semantic analysis (Landauer and Dumais, 1997). We argue that these findings suggest that this semantic space is somewhat representative of the underlying representation of concepts.

While the findings here are promising more can be done to improve the current semantic space. The 12 predictors chosen do not constitute an exhaustive list of potentially relevant dimensions. The sensation dimension, for instance, could be broken up into several dimensions (Visual, Auditory, etc.), which might lead to greater differentiation across more concrete concepts. The inclusion of greater dimensionality would also help decrease the amount of unexplained variance in the model, however, this will happen to a smaller and smaller degree as more dimensions are added. Also now that we have shown proof of concept, future work would benefit from expanding the concepts across grammatical class and concreteness (e.g., more middling concreteness concepts) as this will likely create a semantic space which is more ecologically valid.

Overall, this topographic approach also readily lends itself to computational investigations whereby particular dimensions (e.g., magnitude) or individual clusters (e.g., high emotion, low magnitude) might be selectively lesioned as functions of regional brain damage. Much of the utility of this approach will depend on specifying the nature and fluidity of the topography.

### Conflict of interest statement

The authors declare that the research was conducted in the absence of any commercial or financial relationships that could be construed as a potential conflict of interest.
