# Supplementary material for: Clustering, hierarchical organization, and the topography of abstract and concrete nouns
Source: Front Psychol. 2014 Apr 28;5:360. doi: 10.3389/fpsyg.2014.00360 (PMC4009417; doi:10.3389/fpsyg.2014.00360)
Supplement: Supplementary file 1 [file DataSheet1.DOCX]

Appendix A

Likert Scale Anchor Points


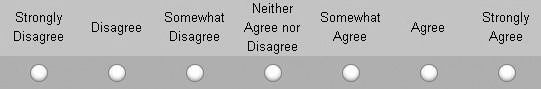


Appendix B

Cluster Membership

| C1 | | C2 | C3 | C4 | | C5 | C6 |
| --- | --- | --- | --- | --- | --- | --- | --- |
| Alligator | Laundry | Apple | Bridge | Autumn | Hospital | Accumulation | Exclusion |
| Ambulance | Lemon | Bird | City | Ball | Hurricane | Acquisition | Fallacy |
| Ankle | Leopard | Blood | Cliff | Bed | Jacket | Addition | Heresy |
| Arrow | Lettuce | Butterfly | Forest | Beverage | Lightning | Amplitude | Idiom |
| Banana | Lion | Candy | Hotel | Boat | Mattress | Appointment | Impossibility |
| Beaver | Lizard | Coffee | Island | Bottle | Menu | Aspect | Irony |
| Beetle | Lobster | Dentist | Lake | Boulder | Money | Availability | Metaphor |
| Bell | Macaroni | Eagle | Landscape | Cake | Moonlight | Brevity | Pretense |
| Blade | Microscope | Gun | Location | Calendar | Neck | Calculation |  |
| Bracelet | Missile | Lip | Mansion | Car | Orchestra | Capacity |  |
| Bubble | Mosquito | Pillow | Mountain | Ceiling | Oven | Category |  |
| Bullet | Mustard | Policeman | Ocean | Chair | Pie | Clearance |  |
| Butter | Necklace | Salad | Palace | Church | Rainbow | Combination |  |
| Caterpillar | Newspaper | Skin | Pond | Clothing | Refrigerator | Convergence |  |
| Chicken | Onion | Sugar | Pyramid | Cottage | Rocket | Deduction |  |
| Chipmunk | Opera |  | River | Desk | Roof | Dimension |  |
| Cocktail | Orchid |  | Road | Diamond | Saloon | Distribution |  |
| Coffin | Peach |  | Sky | Door | Shirt | Duration |  |
| corn | Pig |  | University | Elephant | Snow | Dynasty |  |
| Corpse | Pigeon |  | Volcano | Factory | Sofa | Emergence |  |
| Coupon | Pimple |  | Zoo | Fireplace | Tree | Episode |  |
| Cow | Potato |  |  | Fog | Truck | Establishment | |
| Cranberry | Propeller |  |  | Fountain | Wallet | Extent |  |
| Crocodile | Queen |  |  | Grass | Window | Hierarchy |  |
| Crown | Rabbit |  |  | Hat | Winter | Magnitude |  |
| Cucumber | Raspberry |  |  |  |  | Majority |  |
| Cup | Robin |  |  |  |  | Midnight |  |
| Darkness | Sandal |  |  |  |  | Occasion |  |
| Dove | Shark |  |  |  |  | Origin |  |
| Drum | Shrimp |  |  |  |  | Paradigm |  |
| Eyeball | Skull |  |  |  |  | Proportion |  |
| Fisherman | Skunk |  |  |  |  | Reduction |  |
| Flask | Snake |  |  |  |  | Retention |  |
| Football | Spider |  |  |  |  | Unit |  |
| Frog | Stapler |  |  |  |  | Variety |  |
| Gorilla | Tennis |  |  |  |  |  |  |
| Grasshopper | Thorn |  |  |  |  |  |  |
| Helmet | Toilet |  |  |  |  |  |  |
| Horse | Tomato |  |  |  |  |  |  |
| Item | Tongue |  |  |  |  |  |  |
| Jewel | Tool |  |  |  |  |  |  |
| Key | Towel |  |  |  |  |  |  |
| Kite | Typewriter | |  |  |  |  |  |
| Lamb | Wolf |  |  |  |  |  |  |
| Lamp |  |  |  |  |  |  |  |

| C7 | | C8 | C9 | C10 | C11 | C12 |
| --- | --- | --- | --- | --- | --- | --- |
| Advantage | Myth | Artist | Cowardice | Admiration | Ability | Abundance |
| Adversity | Necessity | Baby | Criticism | Attitude | Accomplishment | Ambition |
| Announcement | Opposition | Body | Deceit | Behavior | Belief | Arrangement |
| Assistance | Originality | Boy | Disagreement | Benefactor | Brain | Attention |
| Assumption | Permission | Cat | Hatred | Character | Faith | Awareness |
| Circumstance | Phenomenon | Child | Malice | Charity | Freedom | Comparison |
| Cognition | Philosophy | Chocolate | Mercy | Confidence | Holiday | Competence |
| Coincidence | Preference | Christmas | Revenge | Crisis | Idea | Comprehension |
| Complication | Preparation | Dad |  | Danger | Improvement | Conclusion |
| Consideration | Recognition | Doctor |  | Duty | Information | Consequence |
| Consistency | Regulation | Father |  | Equality | Intelligence | Corporation |
| Context | reinforcement | Food |  | Expression | Justice | Debt |
| Definition | Replacement | Girl |  | Fantasy | Knowledge | Decision |
| Denial | requirement | Grandmother | | Honesty | Leadership | Democracy |
| Description | Response | Heart |  | Identity | Memory | Determination |
| Destiny | Selection | Kitten |  | Independence | Opportunity | Disaster |
| Development | Separation | Laughter |  | Insight | Reputation | Economy |
| Difference | Situation | Mother |  | Instinct | Responsibility | Event |
| Dilemma | Stimulus | Photo |  | Integrity | Skill | Evidence |
| Distraction | Stupidity | Puppy |  | Interaction | Wisdom | Fact |
| Diversity | Tendency | Sister |  | Kindness |  | Importance |
| Error | Theory | Television |  | Opinion |  | Incentive |
| Example | Topic | Woman |  | Purpose |  | Intention |
| Exception | Translation | |  | Satisfaction |  | Introduction |
| Explanation | Uncertainty | |  | Tradition |  | Logic |
| Gender | Unity |  |  | Truth |  | Miracle |
| Identification | Willingness | |  |  |  | Moment |
| Ignorance |  |  |  |  |  | Occupation |
| Immortality |  |  |  |  |  | Outcome |
| Impairment |  |  |  |  |  | Ownership |
| Incident |  |  |  |  |  | Perception |
| Interruption |  |  |  |  |  | Possibility |
| Legality |  |  |  |  |  | Prediction |
| Limitation |  |  |  |  |  | Presence |
| Mastery |  |  |  |  |  | Production |
| Method |  |  |  |  |  | Promotion |
| Mystery |  |  |  |  |  | Proof |
|  |  |  |  |  |  | Quality |
|  |  |  |  |  |  | Reality |
|  |  |  |  |  |  | Significance |
|  |  |  |  |  |  | Vocabulary |

Appendix C

Removal per Criterion

| Criterion 1 | Criterion 2 | Criterion 3 |
| --- | --- | --- |
| 104 | 94 | 20 |
